# Synthesis of Pt/K_2_CO_3_/MgAlO_x_–reduced graphene oxide hybrids as promising NO_x_ storage–reduction catalysts with superior catalytic performance

**DOI:** 10.1038/srep42862

**Published:** 2017-02-16

**Authors:** Xueyi Mei, Qinghua Yan, Peng Lu, Junya Wang, Yuhan Cui, Yu Nie, Ahmad Umar, Qiang Wang

**Affiliations:** 1 College of Environmental Science and Engineering, Beijing Forestry University, 35 Qinghua East Road, Haidian District, Beijing 100083, P. R. China; 2 Faculty of Environmental Science and Engineering, Kunming University of Science and Technology, Kunming, 650500, Yunnan, P. R. China; 3 Department of Chemistry, College of Science and Arts, Najran University, Najran–11001, Kingdom of Saudi Arabia; 4 Promising Centre for Sensors and Electronic Devices (PCSED), Najran University, Najran–11001, Kingdom of Saudi Arabia

## Abstract

Pt/K_2_CO_3_/MgAlO_x_–reduced graphene oxide (Pt/K/MgAlO_x_–rGO) hybrids were synthesized, characterized and tested as a promising NO_x_ storage and reduction (NSR) catalyst. Mg–Al layered double hydroxides (LDHs) were grown on rGO via *in situ* hydrothermal crystallization. The structure and morphology of samples were thoroughly characterized using various techniques. Isothermal NO_x_ adsorption tests indicated that MgAlO_x_–rGO hybrid exhibited better NO_x_ trapping performance than MgAlO_x_, from 0.44 to 0.61 mmol · g^−1^, which can be attributed to the enhanced particle dispersion and stabilization. In addition, a series of MgAlO_x_–rGO loaded with 2 wt% Pt and different loadings (5, 10, 15, and 20 wt%) of K_2_CO_3_ (denoted as Pt/K/MgAlO_x_–rGO) were obtained by sequential impregnation. The influence of 5% H_2_O on the NO_x_ storage capacity of MgAlO_x_–rGO loaded with 2 wt% Pt and 10% K_2_CO_3_ (2Pt/10 K/MgAlO_x_–rGO) catalyst was also evaluated. In all, the 2Pt/10 K/MgAlO_x_–rGO catalyst not only exhibited high thermal stability and NO_x_ storage capacity of 1.12 mmol · g^−1^, but also possessed excellent H_2_O resistance and lean–rich cycling performance, with an overall 78.4% of NO_x_ removal. This work provided a new scheme for the preparation of highly dispersed MgAlO_x_–rGO hybrid based NSR catalysts.

Nitrous oxides (NO_x_) are known as hazardous compounds and one of the main causes for the formation of highly toxic photochemistry smog and acid rain^1^,^2^,^3^. For the NO_x_ emission control from automobile exhausts, three–way catalysts are highly efficient when the engines work at stoichiometric air/fuel ratio (A/F = 14.7). However, their efficiency diminishes severely in the presence of an excess of oxygen^4^,^5^,^6^,^7^,^8^. Thus, up to now, the NO_x_ abatement for lean–burn engine emissions is still widely recognized as one of the most challenging problems^9^,^10^,^11^.

The NO_x_ storage and reduction (NSR) catalytic treatment appears to be a promising approach to remove NO_x_ under the excess oxygen condition^12^,^13^. The NSR catalysts generally consist of three major components: precious metal (e.g. Pt, Pd, or Rh), alkali or alkaline earth metal oxide as NO_x_ storage material (e.g. Ba, Sr, Ca, Li, K, or Na), and a high surface area support like γ–Al_2_O_3_^14^,^15^,^16^,^17^. During lean conditions, NO_x_ was stored as nitrates (or nitrite), while during the short rich conditions, the stored NO_x_ will be released and then selectively reduced to N_2_^18^,^19^,^20^. Up to date, several groups of NSR catalysts have been developed^21^. However, each type of catalysts have their own shortcomings, such as the narrow range of operating temperatures, thermal degradation and deactivation by sulfur adsorption, etc refs 22, 23. For these reasons, extensive efforts are still being made to develop novel NSR catalysts.

Recently, well mixed transition–metal oxides derived from layered double hydroxides (LDHs) have been suggested to offer potential advantages over Pt/BaO/Al_2_O_3_ in NO_x_ storage–reduction and assessed to be the new generation of NSR catalysts^24^,^25^. LDH is a class of anionic clays^25^. The lamellar structure of LDH is based on positively charged brucite–like sheets with anions and water molecules intercalated between the layers^26^,^27^. For a long time, Mg–Al LDHs, which can be precursors to Mg–Al mixed oxides (MgAlO_x_) have received significant attention for NO_x_ adsorption^24^,^28^,^29^,^30^. Takahashi *et al*.^28^ reported that the NO_x_ storage material could be improved at high^28^,^29^,^30^ temperature by using hydrotalcite–derived MgAl_2_O_4_ spinel as support. Especially, K–supported MgAlO_x_ (K/MgAlO_x_) showed improved NO_x_ adsorption. Park *et al*^24^. reported the Pt and K promoted hydrotalcite–based catalyst displayed better adsorption capacity, the NO_x_ storage capacity of which could be significantly increased to nearly 0.65 mmol · g^−1^, by using 20% CH_3_COOK as the precursors. Li *et al*.^31^ also found that the addition of K enhances the NO_x_ storage capacity of hydrotalcite–derived MnMgAlO catalysts to a great extent compared with the corresponding K–free catalysts, from 0.27 to 0.92 mmol · g^−1^, by using 20% KNO_3_ as the precursor.

Recently, the growth of LDHs on various substrates has received considerable attention^32^. Particularly, graphene structures which possess huge surface area and can be made free–standing could provide a large number of actives sites for adsorption purpose^33^. Luckily, due to the negatively charged nature of graphene and positively charged nature of LDHs, the combination between these two 2D building blocks can be easily achieved by electrostatic interaction^34^. Various strategies have been used to fabricate LDH–GO hybrid materials, including directly mixing LDHs and GO^35^, layer–by–layer self–assembly^36^, and directly LDH formation on GO^32^. These LDH–GO hybrid materials have been used in supercapacitors^32^,^35^,^36^, biosensors^37^,^38^, water treatment^39^, and CO_2_ capture, etc refs 40, 41.

In this contribution, a novel NSR catalyst Pt/K/MgAlO_x_–rGO with good NO_x_ storage capacity and excellent lean–rich cycling stability was developed by *in situ* grown of Mg–Al LDHs on rGO and the subsequent sequential impregnation of Pt and K_2_CO_3_. The influence of the addition of rGO on the NO_x_ storage capacity of MgAlO_x_ based NSR catalysts was investigated. All samples were thoroughly characterized by XRD, XPS, SEM, TEM, FT–IR, Raman and BET analyses. The influences of Pt and K doping on the NO_x_ storage capacity of MgAlO_x_–GO hybrids were also evaluated. Finally, the NO_x_ storage and reduction cycling performance and thermal stability of 2Pt/10 K/MgAlO_x_–GO hybrid catalyst were also tested.

## Results and Discussion

**Figure 1:**
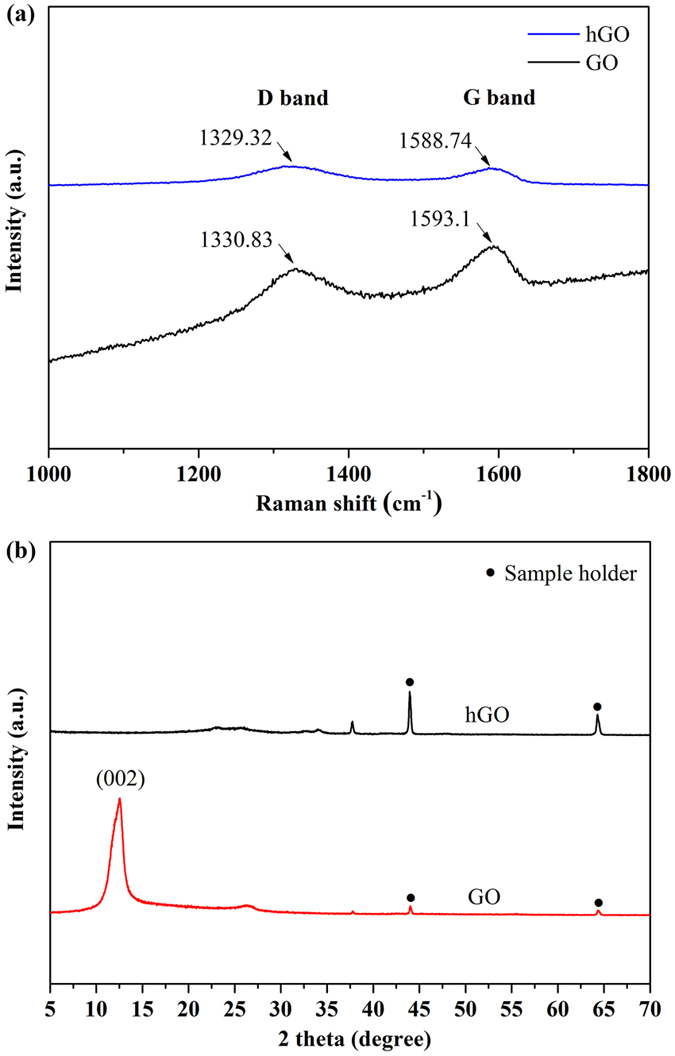
(**a**) Raman patterns of GO and hGO and (**b**) XRD patterns of GO and hGO.

Figure 1(a) shows the Raman spectra of GO and the GO treated by hydrothermal method at 120 °C under pH = 10 for 12 h (designated as hGO). It is well known that graphene exhibits two main characteristic peaks: the D band at ~1350 cm^−1^, arising from a breathing mode of k–point photons of A_1g_ symmetry and the G band at ~1575 cm^−1^, arising from the first order scattering of E_2g_ phonon of sp^2^ C atoms^42^. In our present study, it can be seen that both GO and hGO exhibited a D band at 1330 cm^−1^ and a G band at 1593 cm^−1^. It is also found that the hGO showed a relatively higher intensity of D to G band (1.01) than that of GO (0.93). These observations confirmed the formation of new graphitic domains after the hydrothermal treatment process^43^. The XRD patterns of dried GO and hGO were also shown in Fig. 1(b). As it is expected that the dried GO displayed a layered structure with an interlayer spacing of 0.86 nm corresponding to the feature diffraction peak at 10.2°, revealing the introduction of oxygen functional groups on the graphite sheets^44^,^45^. However, after treated with hydrothermal method under pH = 10, the hGO exhibited the major peak at about 23–24°. This gives an interlayer spacing of approximately 0.37–0.38 nm. This interlayer spacing is much smaller than that for GO (0.86 nm), and is closer to the (002) graphite peak of 0.336 nm^46^. Taken together, both Raman and XRD data suggested the evidence to support the formation of rGO under hydrothermal condition at pH = 10. Therefore, the obtained hybrid material was denoted as LDH/rGO.

**Figure 2:**
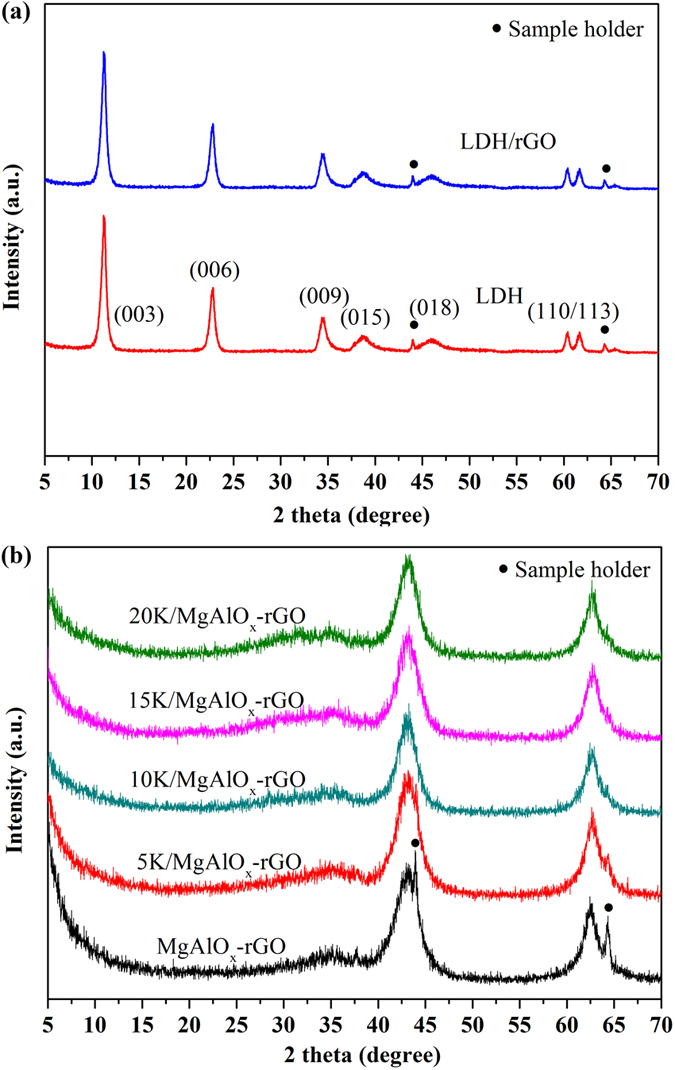
(**a**) XRD patterns of neat LDH, and LDH/rGO hybrid, and (**b**) XRD patterns of XK/MgAlO_x_–rGO hybrids (X = 5, 10, 15, and 20 wt%).

The XRD patterns of neat LDH, and the as synthesized LDH/rGO are shown in Fig. 2(a). Neat LDH exhibited a hexagonal structure with sharp symmetric peaks for the (*003*), (*006*) and (*009*) planes and broad asymmetric peaks for (*015*) and (*018*) planes which are characteristic of hydrotalcites. The diffraction lines at about 60.5° and 61.5° are ascribed to the (*110*) and (*113*) reflections^47^. In the case of LDH/rGO, the diffraction peaks are similar to those of pristine LDH, but no shift in basal reflection peaks was observed. This suggests the similarity in the intercalation of 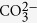 anions into the layer galleries between the pristine LDH and the distributed LDH grown on rGO^37^. Figure 2(b) shows the XRD patterns of K_2_CO_3_ doped MgAlO_x_–rGO (K/MgAlO_x_–rGO) samples with different loadings of K_2_CO_3_. These diffractograms showed two intense lines (43°, and 62.5°), which are typical to MgO–like phase or likely magnesia–alumina solid solution (JCPDS 45–946). No diffraction peaks corresponding to K–related species were observed. Many similar results have been reported that the K–containing species could hardly be recognized by XRD analysis^48^,^49^,^50^,^51^. So, it was deduced that the K_2_CO_3_ species were well dispersed on the support and probably existed in amorphous phase that may be below the XRD detection limit^48^.

**Figure 3:**
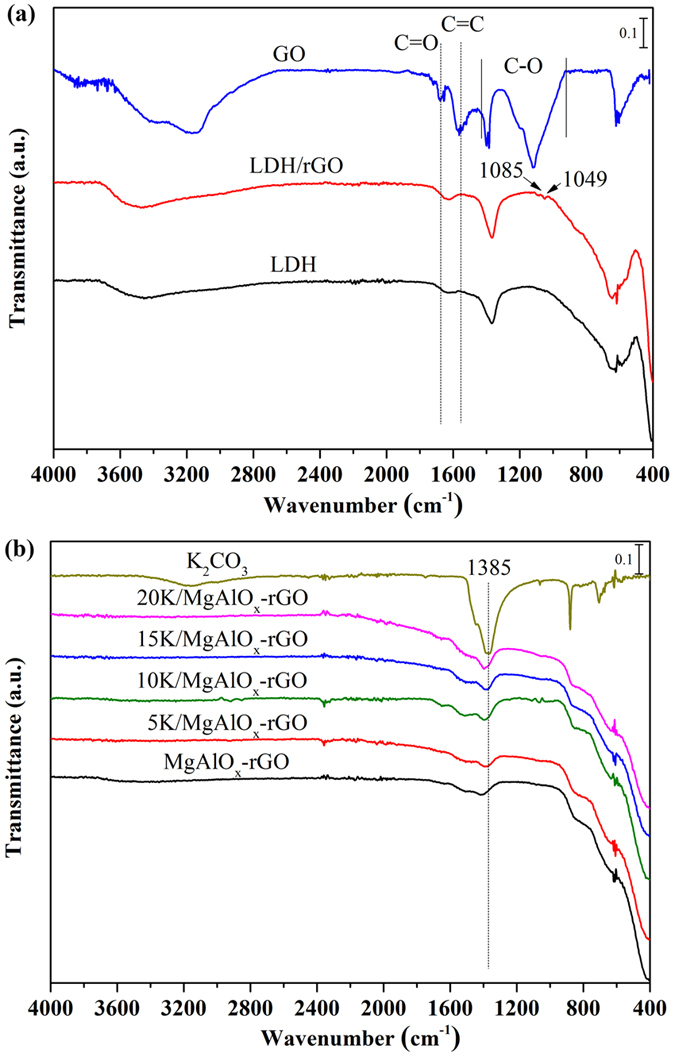
(**a**) ATR FT–IR spectra of GO, LDH, and LDH/rGO hybrids, and (**b**) ATR FT–IR spectra of XK/MgAlO_x_–rGO (X = 5, 10, 15, and 20 wt%).

ATR‒FTIR spectra of dried GO, neat LDH, and LDH/rGO hybrids are shown in Fig. 3(a). The absorption peaks at 3400 cm^−1^ for the GO correspond to the stretching mode of O–H. The characteristic features for GO are the stretching vibrations of C=O (carboxylic acid) at 1677 cm^−1^, C–O–C (epoxy) and C–O (alkoxy) at 925–1445 cm^−1^, and C=C at 1575 cm^−1^, originating from the skeletal vibrations of un–oxidized graphitic domains^52^,^53^,^54^,^55^,^56^. In all the spectra of neat LDH samples, a strong broad vibration band in the 3200 to 3600 cm^−1^ range can be observed, which can be attributed to the O–H stretching vibration of water molecules and hydroxyl groups that belong to brucite layers. The band at 1624 cm^−1^ was due to the H–O–H bending vibration. A strong band at 1360 cm^–1^ was attributed to the carbonate group. The bands below 800 cm^−1^ were ascribed to Al–O and Mg–O vibrations^57^. When combined, the peaks in the spectra of LDH/rGO are consistent with those of the LDH and GO, indicating the successful hybridization of rGO with LDH. As no information about K species was given by XRD results, thus ATR FT–IR technique was employed to investigate the states of K species, as shown in Fig. 3(b). The bulk K_2_CO_3_ used as the precursor of potassium salt shows the characteristic IR bands in the region of 1800–1000 cm^−1^. When K was introduced, the K_2_CO_3_ species was clearly detected, with the characteristic peak located at 1385 cm^−1 ^58. The intensity of this characteristic peak became stronger with the increase in K_2_CO_3_ loading.

The BET specific surface areas of MgAlO_x_, MgAlO_x_–rGO, 10 K/MgAlO_x_, and 10 K/MgAlO_x_–rGO were also investigated. The specific surface area of MgAlO_x_–rGO hybrid was 230.6 m^2^ · g^−1^, which was larger than that of neat MgAlO_x_ (207.5 m^2^ · g^−1^). It has been proven that the addition of rGO can decrease the aggregation of MgAlO_x_, resulting in more exposed MgAlO_x_ nanosheets and enlarged specific surface area^59^. After loading K_2_CO_3_, the specific surface area decreased a little for both 10 K/MgAlO_x_ (199.1 m^2^ · g^−1^) and 10 K/MgAlO_x_–rGO (129.2 m^2^ · g^−1^), probably due to the pore blocking by K_2_CO_3_.

**Figure 4:**
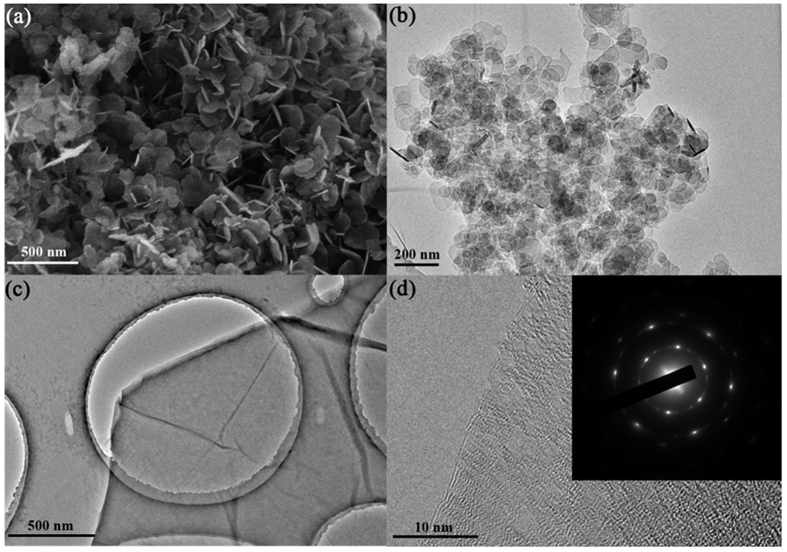
(**a**) FE–SEM of Mg–Al–CO_3_ LDH, (**b**) HR–TEM image of Mg–Al–CO_3_ LDH, (**c**) HR–TEM image of graphene oxide, and (**d**) HR–TEM image of graphene oxide with higher resolution, the inset shows SAED pattern of the GO.

The morphologies and structures of the GO and LDH were investigated by XRD and HR–TEM, as shown in Fig. 4. The SEM image of pure LDH (Fig. 4(a)) shows that the sample consist of a large number of LDH nanoplates, which have aggregated in a disordered fashion with a definite shape. The image in Fig. 4(b) shows that the pure LDH consists of abundant particles with a definite shape, which was of high quality in terms of crystallinity, and the mean size is determined to be ca. 100–200 nm. However, because of the high surface energy and strong van der Waals interactions, aggregated LDHs were observed. Figure 4(c) reveals that the GO nanosheets are curled and wrinkled just like a transparent corrugated voile due to the intrinsic nature of the GO nanosheets^60^. Closer observation of the HR–TEM image (Fig. 4(d)) shows the fringes at the edge of a flake, indicating the stacking of 2–3 monolayers. The inset SAED pattern of the GO shows the typical hexagonal crystal lattice which has a six–fold symmetry.

**Figure 5:**
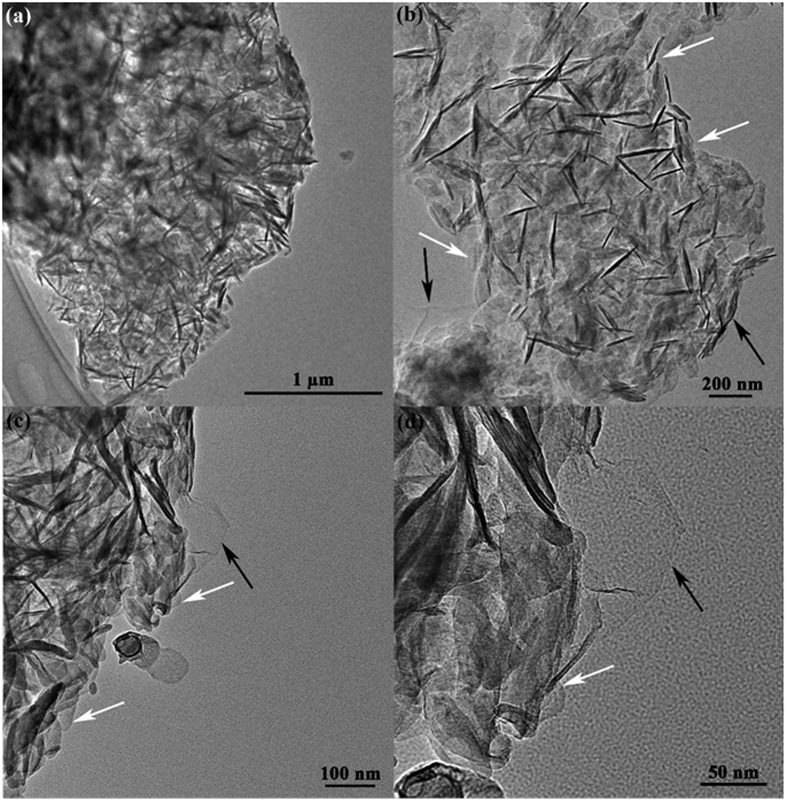
HR–TEM images of the LDH/rGO hybrids with different magnifications, (**a**) ×5000, (**b**) ×20000, (**c**) ×15000, and (**d**) ×15000.

The LDH platelets are adsorbed on the surface of rGO nanosheets due to the electrostatic interaction between rGO and LDH^61^. Both LDH and rGO nanosheets were clearly observed was showed in Fig. 5, as indicated by the white arrows and black arrows, respectively. Form Fig. 5(b), it is clear that the LDH/rGO nanoplates are distributed on the surface of rGO nanosheets. Some LDH grew with *ab*–planes of the crystallites parallel to rGO, whereas some LDH grew with *ab*–planes perpendicular to rGO surface. Figure 5(c) reveals a marked contrast between the LDH nanoparticles and the underlying rGO. The thickness and the average particle size of the LDH nanoparticles were found to be ca. 10 and 50 nm, respectively. At higher magnification (Fig. 5(d)), it can be seen more clearly. The distribution of the LDH is not uniform because the rGO sheets capture the LDH mainly via reactive groups, and the active sites of rGO are not homogeneously distributed on the nanosheets^32^.

**Figure 6:**
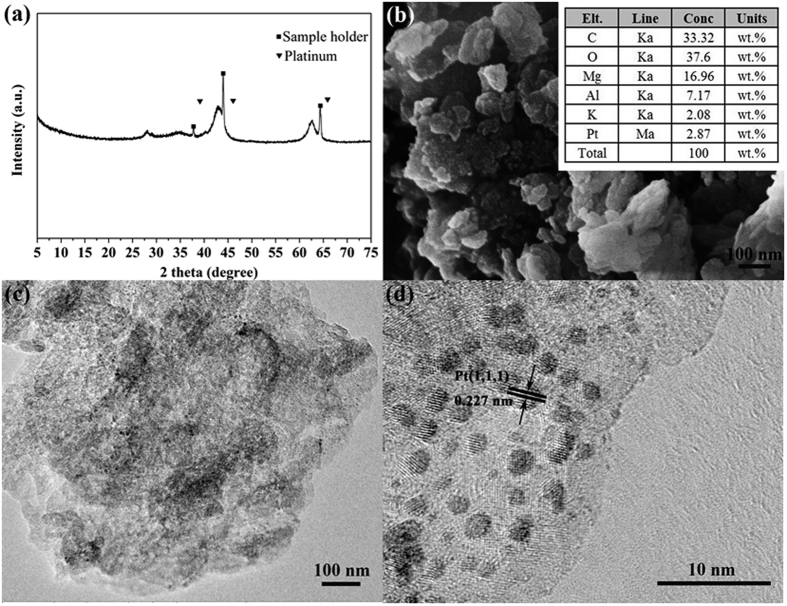
(**a**) XRD patterns of 2Pt/10 K/MgAlO_x_–rGO catalyst, (**b**) SEM–EDS analyses of 2Pt/10 K/MgAlO_x_–rGO catalyst, (**c**) TEM image of 2Pt/10 K/MgAlO_x_–rGO catalyst, (**d**) TEM image of 2Pt/10 K/MgAlO_x_–rGO catalyst with higher resolution.

As noble metals, such as Pt, plays a key role in the NO_x_ storage and reduction cycles, it is of great interest to investigate its existence in detail. No relevant changes are introduced in the diffraction patterns with Pt incorporation (Fig. 6(a)), which might due to the high dispersion of Pt in the catalyst^62^. To further elucidate the existence of Pt, SEM–EDS was performed on the catalyst, and the images are displayed in Fig. 6(b). The SEM image is fully consistent with the XRD results. No Pt–containing crystals could be observed, suggesting Pt species that are so small and highly dispersed. However, the EDS analysis confirmed the existence of Pt with an average Pt loading of 2.87 ± 0.38 wt%. This value is somehow higher than that of the theoretical amount used for the preparation, which can be attributed to the detection limitation of EDS. Figure 6(c) and (d) show the HR–TEM images of the catalyst. In all cases the Pt nanoparticles are dispersed on the support surface (Fig. 6(c)), with an average of particle size of 2.7–3.5 nm being determined from the HR–TEM images (Fig. 5(d)). The d–spacing value of 0.227 nm coincides with that of fcc Pt (111)^63^.

**Figure 7:**
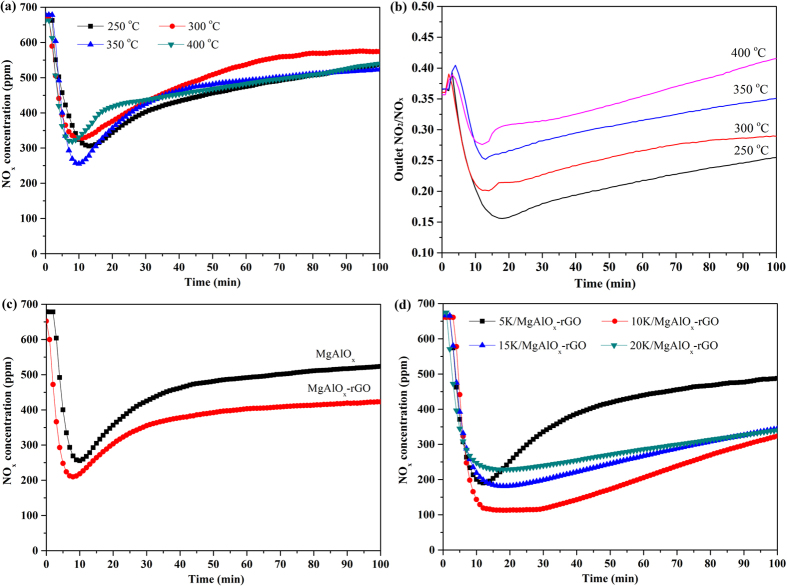
(**a**) Isothermal NO_x_ storage over MgAlO_x_ at 250, 300, 350, and 400 °C, (**b**) Outlet NO_2_/NO_x_ over MgAlO_x_ at 250, 300, 350, and 400 °C, (**c**) isothermal NO_x_ storage over MgAlO_x_ and MgAlO_x_–rGO at 350 °C, (**d**) isothermal NO_x_ storage over XK/MgAlO_x_–rGO (X = 5, 10, 15, and 20 wt%) at 350 °C.

The NO_x_ storage amount on MgAlO_x_ was first measured by the isothermal storage of NO+O_2_ at 250, 300, 350, and 400 °C, respectively, as shown in Fig. 7(a). The NO_x_ concentration in the outlets first showed a sharp decrease during the first several min and then followed by a gradual increase as a function of time. The lowest values were reached after about 10–15 min for all temperature range (250–400 °C). The difference between inlet and outlet NO_x_ concentration corresponded to the NO_x_ stored on the catalyst. It took around 100 min for MgAlO_x_ to become saturated. Particularly, the sample can quickly capture the NO_x_ within the first ca. 10 min, reaching the lowest point (~250 ppm) at 350 °C. Upon calculation, a maximum value of adsorbed NO_x_ species close to 0.44 mmol · g^−1^ catalyst was achieved at 350 °C. Since the reaction time was only 100 min and the reaction balance has not been reached yet. Both NO_2_ storage and NO conversion to NO_2_ occurred at the same time, therefore the NO conversion to NO_2_ could not be calculated accurately. But for MgAlO_x_, the NO to NO_2_ conversion could be approximately estimated by the outlet NO_2_/NO_x_ value because the reaction was close to equilibrium. As shown in Fig. 7(b), it is clear that the conversion increased with the increase in testing temperature from 250 to 400 °C. However, pure MgAlO_x_ without noble metal such as Pt showed low NO oxidation activity.

In our previous work, the addition of 7 wt% GO showed better NO_x_ storage capacity due to decreasing the aggregation of LDO and resulting in more exposed LDO nanosheets^62^. Therefore, in this study rGO was also investigated as a supporting material to optimized the NO_x_ storage capacity of neat LDH. Figure 7(c) exhibits the isothermal NO_x_ storage performance of MgAlO_x_ and MgAlO_x_–rGO at 350 °C. For MgAlO_x_–rGO hybrid, it exhibited quicker NO_x_ uptake and higher NO_x_ capture capacity than those of MgAlO_x_. The NO_x_ adsorption rate is very high and the reachable lowest NO_x_ concentration was about 200 ppm. A maximum NO_x_ storage value around 0.61 mmol·g^−1^ was obtained, even much higher than that of MgAlO_x_ (0.44 mmol · g^−1^). Therefore, these data suggest that the absolute NO_x_ storage capacity of MgAlO_x_ can be markedly improved by adding 7 wt% GO, which was used in the hydrothermal process for its functional surfaces, worked as a template for the nucleation of Mg–Al LDHs and provided anchoring sites to the LDH nanosheets^64^. For LDH, the addition of K could remarkably improve their performance for NO_x_ storage^28^,^65^.

The presence of K increased the mobility of surface oxygen species on the catalysts^66^ and enhanced the formation of low melting–point compounds^67^. Therefore, the influence of K doping has been investigated over MgAlO_x_–rGO at 350 °C, as shown in Fig. 7(d). In this contribution, K_2_CO_3_ was regarded as the precursor material. For the samples with increasing the loading of K_2_CO_3_, the overall amount of NO_x_ stored was enhanced significantly and the reachable lowest NO_x_ concentration was about 100 ppm when the K loading was increased to 10 wt%. Meanwhile, the NO_x_ storage became a rather slow process and the highest NO_x_ storage amount was improved to 0.81 mmol · g^−1^. However, the additional K did not provide further enhancement in the amount of stored NO_x_. At K loading of 15 and 20 wt%, the overall NO_x_ storage activity decreased slightly. The presence of more bulk or bulk–like K_2_CO_3_ in these samples makes NO_x_ storage more difficult due to the gradually increased surface to bulk diffusion resistance. Finally, the NO_x_ storage amount of neat MgAlO_x_ can be significantly promoted from 0.44 to 0.81 mmol · g^−1^ with the addition of 7 wt% GO and 10 wt% K_2_CO_3_.

**Figure 8:**
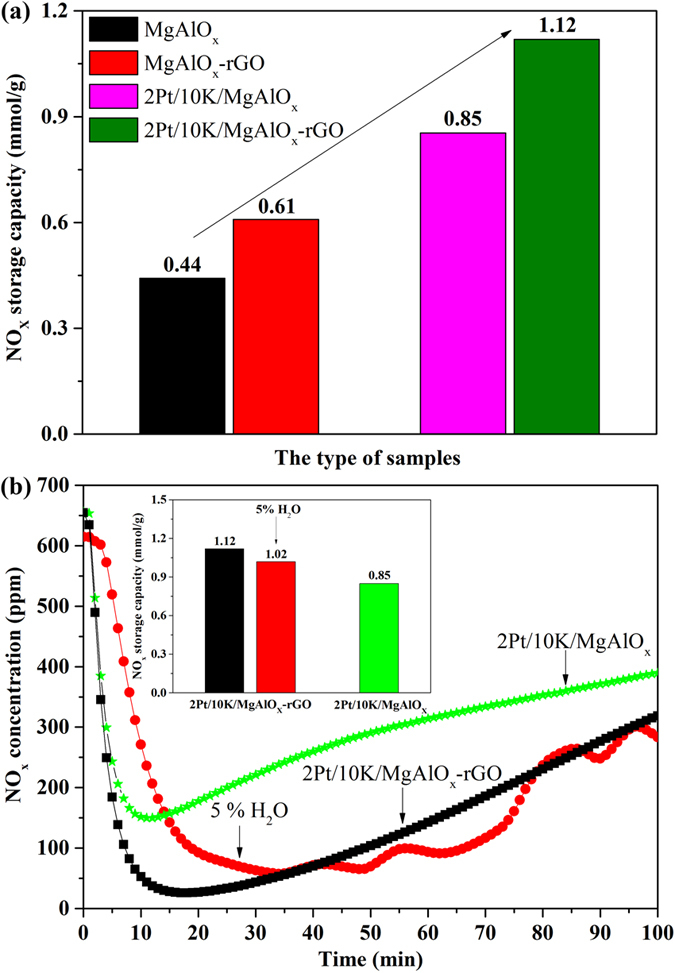
(**a**) Isothermal NO_x_ storage over 2Pt/10 K/MgAlO_x_, 2Pt/10 K/MgAlO_x_–rGO catalysts and the influence of 5% H_2_O on 2Pt/10 K/MgAlO_x_–rGO catalysts tested at 350 °C. (**b**)The NO_x_ storage capacities of MgAlO_x_, MgAlO_x_–rGO, 2Pt/10 K/MgAlO_x_, and 2Pt/10 K/MgAlO_x_–rGO catalysts tested at 350 °C.

Precious metals represent an obvious choice for both the NO oxidation to NO_2_ during lean condition and the adsorbed NO_x_ reduction during rich condition for the NSR catalysts, due to their unparalleled red–ox activity, and Pt has been the primary oxidation catalyst choice for NSR samples to date. Thus, the influence of Pt doping on 10 K/MgAlO_x_ and 10 K/MgAlO_x_–rGO hybrids was also studied, as shown in Fig. 8(a). In comparison to Pt unloaded samples, all Pt loaded samples show much higher NO_x_ storage activity at 350 °C, which was increased from 0.44 and 0.61 mmol · g^−1^ to 0.85 and 1.12 mmol · g^−1^, respectively. This improvement in NO_x_ storage capacity can be ascribed to the promoted NO oxidation to NO_2_ by Pt, and highly dispersed Pt in the catalyst. Moreover, the containing Mg would help Pt to be well dispersed on the supports, leading to the increase in NO_x_ storage performance^8^. Figure S1 further confirmed the excellent NO_x_ storage capacity and NO oxidation in obtained 2Pt/10 K/MgAlO_x_–rGO catalyst during the whole reaction through plotting the NO, NO_2_, NO_x_, and the ratio of NO/NO_2_ in the outlet.

H_2_O is one of the main components in the vehicle exhausts and often affects the storage and regeneration performance of NSR catalysts. Previous investigations have shown that the presence of H_2_O affect the NO_x_ storage capacity^68^,^69^. Therefore, the H_2_O poisoning effect on Pt/K/MgAlO_x_–rGO catalyst was then studied. The influence of 5% H_2_O on NO_x_ storage over Pt/K/MgAlO_x_–rGO catalyst at 350 °C is shown in Fig. 8(b). There is an almost complete capture of NO_x_ for both conditions at the beginning. However, the saturation concentration is lower and the stability is higher in the absence of H_2_O. From the inset of Fig. 8(b), it is evident that only slightly less NO_x_ is stored when introduced 5% H_2_O, from 1.12 to 1.02 mmol · g^−1^, which is consistent with previous studies^68^,^69^. The common agreement in the literature is that the water vapor reduces the NO_x_ storage capacity and leads to catalyst deactivation by decreasing the number of available active sites. The results demonstrated that although Pt/K/MgAlO_x_–rGO was also slightly effected by 5% H_2_O, its performance is still much better than that of Pt/K/MgAlO_x._

**Figure 9:**
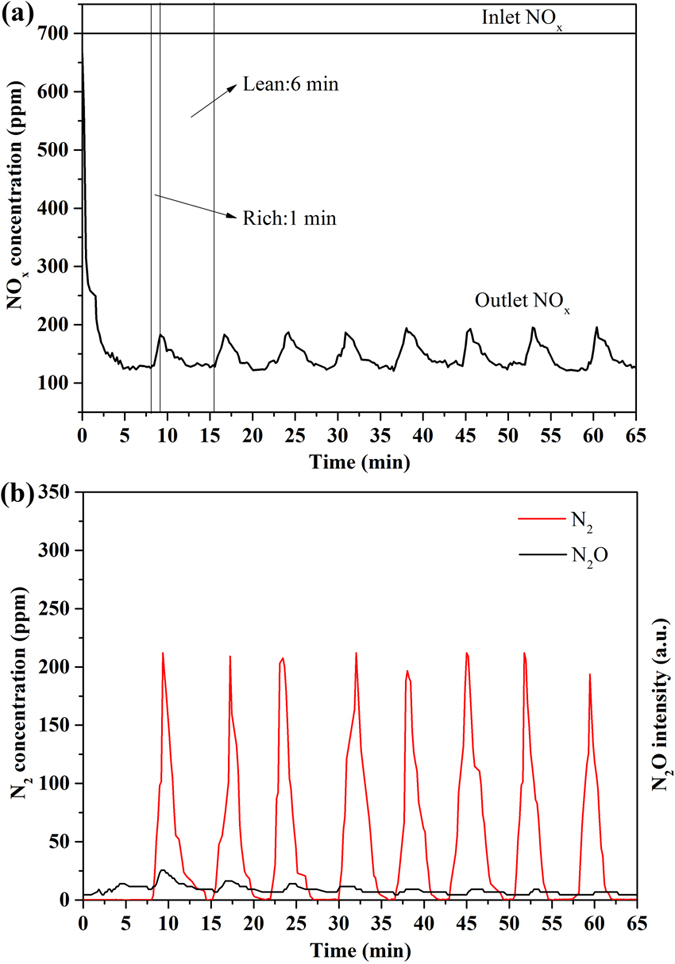
(**a**) Evolution of effluent NO_x_ during lean–rich cycling tests over 2Pt/10 K/MgAlO_x_–rGO catalyst at 350 °C, and (**b**) evolution of effluent N_2_ and N_2_O during lean–rich cycling tests over 2Pt/10 K/MgAlO_x_–rGO catalyst at 350 °C.

Figure 9(a) depicts the NO_x_ concentration (ppm) profiles of the inlet and outlet gases over 2Pt/10 K/MgAlO_x_–rGO catalyst during the NO_x_ storage and reduction cyclic reaction at 350 °C. At beginning of the lean phase (6 min), a sharp NO_x_ removal peak appeared immediately after the 2Pt/10 K/MgAlO_x_–rGO contact was in contact with the gas mixture of NO+O_2_, which indicated a nearly complete adsorption and/or conversion of NO_x_ on the catalyst. The NO_x_ profiles obtained during the lean cycle showed almost the same shape, and the NO_x_ level reached a more or less constant value afterwards. Meanwhile, the NO_x_ concentration decreased slowly with time and reached 150 ppm at the end of the lean phase. This is far below the inlet NO_x_ concentration of 700 ppm, showing a large amount of NO_x_ was stored. When it was switched to the rich phase (1 min), a quick increase in NO_x_ concentration was observed in the first few seconds. This may be attributed to two reasons. One is that the rate of adsorption of NO_x_ decreased when oxygen was cut off. The other is that in the first few seconds of rich phase, a certain amount of oxygen remained on the Pt^70^. Because of the high reductive activity of Pt, the main product was N_2_ (~230 ppm) during rich cycles (Fig. 9(b)). Due to the limit of experimental condition, the N_2_O could not be calibrated accurately. But the intensity of N_2_O was very low compared with N_2_ in the present results (as shown in Fig. S2) and the similar discussion has been reported by other literature^6^,^7^. Overall, more than 78.40% of NO_x_ was removed during the whole lean–rich cycling tests.

**Figure 10:**
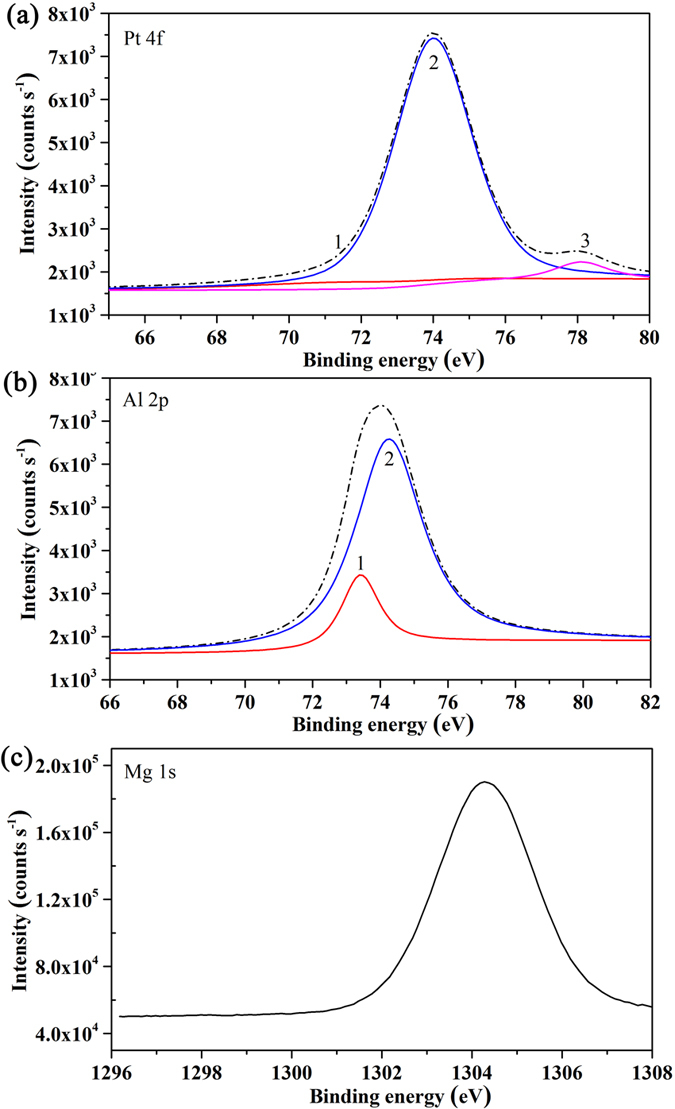
XPS analyses of synthesized 2Pt/10 K/MgAlO_x_–rGO catalysts.

The nature of the active species present on the surface is important for establishing the properties of the catalyst. For this purpose, the XPS analysis has been performed in order to obtain information about the surface composition of the obtained catalysts. Figure 10 shows the typical XPS spectra of 2Pt/10 K/MgAlO_x_–rGO. In Fig. 10(a), the spectrum was deconvoluted into three components labelled as 1, 2 and 3 with respective binding energies of 71.55, 74.0, and 78.16 eV. The relative intensities (%) of the three components were 9.9, 84.7 and 5.4%, respectively. The Pt-4f_7/2_ signal at 71.55 eV can be assigned to zero-valent platinum. The relative shift from the value of 70.8 eV for the bulk metal is probably due to a contribution from metal-support interaction or small cluster-size effects^71^. The signal at 74.0 and 78.1 eV could be ascribed to Pt^2+^ and Pt^4+^ oxidation states^72^. Oxygen chemisorption easily occurs at step and kink sites present on the surface of Pt clusters^73^. For the calcined catalyst, the Al 2p signals labelled as 1 and 2 were adjusted by using two components, as shown in Fig. 10(b). The first one, occurring around at 74.23 eV, is assigned to octahedrally coordinated Al^3+^, whereas the second one, occurring around 73.41 eV must be assigned to tetrahedrally coordinated Al^3+ ^74. The intensity of the octahedrally coordinated Al^3+^ (81.68%) is higher than that of tetrahedrally coordinated Al^3+^ (16.10%). That may be assigned to the “memory effect” of LDH after exposure in the air for a long time^75^. Figure 10(c) displayed the binding energy of Mg 1 s in the obtained catalyst. The peak located around 1304.28 eV must be assigned to Mg^2+^ in periclase MgO structure^76^.

**Figure 11:**
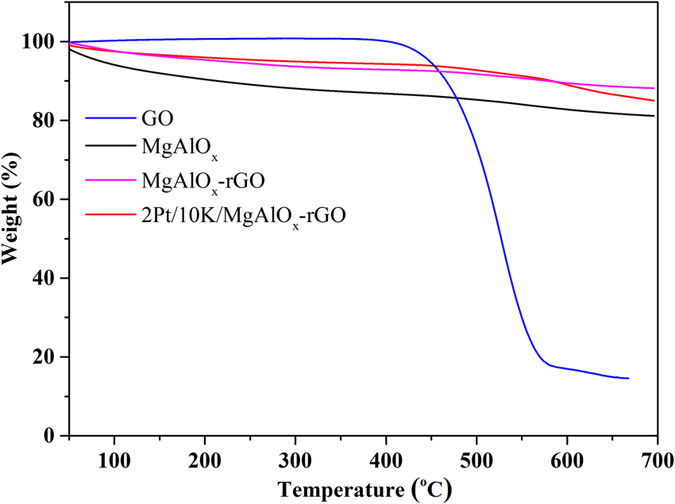
TGA analyses of synthesized GO, MgAlO_x_, MgAlO_x_–rGO, and 2Pt/10 K/MgAlO_x_–rGO catalysts.

The thermal stability of catalyst is another important issue. To investigate this, GO, MgAlO_x_, MgAlO_x_–GO and 2Pt/10 K/MgAlO_x_–GO were tested in the TGA in the temperature range of 50–700 °C and in the presence of air, as shown in Fig. 11. The data indicates that GO is only thermally stable up to 400 °C. When the temperature was higher than 400 °C, GO started to decompose quickly, with a total weight loss of ca. 84.8% at 650 °C. This data suggested that neat GO is not thermally stable. The weight decrease of MgAlO_x_ was only 16.92% in the temperature range of 50–700 °C, which was due to the loss of loosely held water in the interlayer pace^77^. While once GO was hybridized with MgAlO_x_, its thermal stability was significantly improved. Moreover, the 2Pt/10 K/MgAlO_x_–rGO catalyst also showed excellent thermal stability, the decrease of which was approximately 14% in the temperature range of 50–700 °C. There was only a slight decrease in weight with increasing the calcination temperature from 50 to 700 °C. So the thermal stability of the catalyst is not considered as a problem when the operating temperature of this novel catalyst is only ca. 350 °C. Taking its thermal and cycling stability into consideration, 2Pt/10 K/MgAlO_x_–rGO displayed great potential to work as a NSR catalyst with improved NO_x_ storage ability.

## Conclusion

In this contribution, a novel NSR catalyst Pt/K/MgAlO_x_–rGO with good NO_x_ storage capacity and excellent lean–rich cycling stability was developed by *in situ* grown of Mg–Al LDHs on rGO and the subsequent sequential impregnation of Pt and K_2_CO_3_. Mg–Al LDHs were grown on rGO via *in situ* hydrothermal crystallization. The Pt nanoparticles were well dispersed with an average particle size of ca. 2.7–3.5 nm. Isothermal NO_x_ storage tests demonstrated that the optimal operating temperature for MgAlO_x_ was 350 °C. By introducing only 7 wt% of GO, the NO_x_ storage capacity of neat MgAlO_x_ was significantly improved from 0.44 to 0.61 mmol · g^−1^, which can be attributed to the enhanced particle dispersion and stabilization. By impregnating 2 wt% Pt and 10 wt% K_2_CO_3_, the NO_x_ storage capacity was further increased to 1.12 mmol · g^−1^. We also demonstrated that our newly developed 2Pt/10 K/MgAlO_x_–rGO catalyst possesses excellent H_2_O resistance and thermal stability. The obtained Pt/K/MgAlO_x_–rGO catalyst also exhibited excellent lean–rich cycling performance, with an overall 78.4% of NO_x_ removal.

## Methods

### Preparation of Mg–Al–CO_3_ LDH

LDH with Mg/Al = 3 was prepared using a hydrothermal procedure. In the method, 100 mL aqueous salt solution containing a mixture of 0.075 mol Mg(NO_3_)_2_ · 6H_2_O and 0.025 mol Al(NO_3_)_3_ · 9H_2_O was added drop–wise into a 100 mL aqueous solution containing 0.05 mol Na_2_CO_3_. In the meantime, the pH of the precipitation was controlled at 10 using a 4 M NaOH solution. The resulting mixture was hydrothermal treated at 120 °C for 12 h. It was then filtered and washed with deionized water until pH of the wash water became 7, and washed with acetone for several times, followed by drying at 60 °C.

### Preparation of Mg–Al LDH/rGO hybrid

Firstly, the GO used in this study were prepared by modified Hummers method as described in our previous work^62^. The Mg–A1–CO_3_ LDH/rGO hybrids were prepared via a hydrothermal method. In the method, 50 mL aqueous salt solution containing a mixture of 0.0375 mol Mg(NO_3_)_2_·6H_2_O and 0.0125 mol Al(NO_3_)_3_·9H_2_O was added drop‒wise to another 250 mL solution containing 0.025 mol Na_2_CO_3_ and 200 mL GO dispersion (1 g · L^−1^) under vigorous stirring at room temperature. The pH of the mixture was kept constant at 10 by adding a 4 M NaOH solution. The resulting mixture was hydrothermal treated at 120 °C for 12 h. It was then filtered and washed with deionized water until pH of the wash water became 7, and washed with acetone for several times, followed by dring at 60 °C in an oven. The obtained samples were designated as LDH/rGO.

### Preparation of Pt/K/MgAlO_x_–rGO

The Pt/K/MgAlO_x_–rGO catalysts were prepared using a successive impregnation method. LDH/rGO was first pretreated at 400 °C in air for 5 h to obtain MgAlO_x_–rGO hybrids. Next, the obtained MgAlO_x_–rGO support was first impregnated with H_2_PtCl_6_ · 6H_2_O ethanol solution (100 g · L^−1^), dried at 60 °C for overnight, and calcined in air at 400 °C for 5 h. The sample was then impregnated with K_2_CO_3_ ethanediol solution (10 g · L^−1^), followed by drying at 60 °C and calcining at 400 °C for 5 h. The loading of Pt was 2 wt%, and the loading of K_2_CO_3_ was controlled to be 5, 10, 15, and 20 wt%, respectively. The obtained catalysts were denoted as 2Pt/XK/MgAlO_x_–rGO, where 2 and X represent the weight loading of Pt and K_2_CO_3_, respectively. XK/MgAlO_x_–rGO samples were also prepared similarly for comparison.

### Characterization

The phase structure and morphology of the samples were characterized by powder X–ray diffractometer (XRD, Shimadzu XRD–7000) with Cu Kα radiation and a power of 40 kV × 30 mA, X–ray photoelectron spectra (XPS) were recorded on a Thermo escalab 250Xi spectrometer equipped with A1 Kα X–ray source, a scanning electron microscope (FE–SEM, SU–8010), and a high resolution transmission electron microscopy (HR–TEM, JEOL 2010). Raman spectra were obtained on a Renishaw inVia Raman spectrometer with 532 nm wavelength incident laser light. Attenuated total reflectance‒Fourier transform infrared spectroscopy (ATR‒FTIR, Bruker VERTEX 70) was used to identify the functional groups of samples. BET specific surface areas were measured with a physisorption analyzer (SSA–7000, Builder). The weight loss of samples were measured using a Q50 TGA analyser (TA Instruments, N_2_ flow rate = 60 mL · min^−1^) in the temperature range of 50–700 °C.

### NO_x_ storage and reduction reaction

For NO_x_ storage reaction, experiments were carried out in a fixed bed reactor (10 mm internal diameter) under atmospheric pressure. All gases except H_2_O were introduced into the reactor via several mass flow controllers (Brooks Instruments). Liquid water was continuously added using a syringe pump (Lead fluid, TYD01) into a stainless steel tube wrapped with a temperature–controlled heating tape, in which the water vapor was generated. An on–line NO–NO_2_–NO_x_ analyser (Thermo Scientific 42i–HL, USA) was used to measure the concentration of NO, NO_2_ and NO_x_ in the outlet gas. The NO_x_ storage capacity (NSC) was calculated by integrating the concentration curves of NO_x_ when steady state was reached according to the following equation (1).


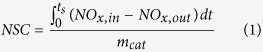


For each test, the time span is about 100 min and 0.2 g sample that was pre–calcined at 400 °C for 5 h in air was employed. Alternative lean/rich cyclic NO_x_ storage and reduction was performed in the same reactor described as above under cyclic operation. Totally 8 cycles of 6 min lean and 1 min rich were applied, and 0.2 g of sample was used to measure the activity. NO_x_ concentrations in both inlet and outlet were measured with an on–line quadrupole mass spectrometer (QGA, Hidden, UK). The mixture gas consisted of a continuous flow of 700 ppm NO_x_ (430 ppm NO and 270 ppm NO_2_), 10% O_2_ (for lean condition only), 3.5% H_2_ (for rich condition only), 5% H_2_O (for evaluating the poisoning effect of H_2_O only) and Ar as the balance gas, with a flow rate of 100 mL · min^−1^ (GHSV = 15000 h^−1^). The reaction temperature was controlled at 250, 300, 350, and 400 °C, respectively.

## Additional Information

**How to cite this article**: Mei, X. *et al*. Synthesis of Pt/K_2_CO_3_/MgAlO_x_–reduced graphene oxide hybrids as promising NO_x_ storage–reduction catalysts with superior catalytic performance. *Sci. Rep.* **7**, 42862; doi: 10.1038/srep42862 (2017).

**Publisher's note:** Springer Nature remains neutral with regard to jurisdictional claims in published maps and institutional affiliations.

## Supplementary Material

Supplementary Information
