# Supplementary material for: Synthesis of Pt/K2CO3/MgAlOx–reduced graphene oxide hybrids as promising NOx storage–reduction catalysts with superior catalytic performance
Source: Sci Rep. 2017 Feb 16;7:42862. doi: 10.1038/srep42862 (PMC5311869; doi:10.1038/srep42862)
Supplement: Supplementary Information [file srep42862-s1.doc]

**Supporting Information**

# Synthesis of Pt/K_2_CO_3_/MgAlO_x_–reduced graphene oxide hybrids as promising NO_x_ storage–reduction catalysts with superior catalytic performance

Xueyi Mei^1^, Qinghua Yan^1^, Peng Lu^1^, Junya Wang^2^, Yuhan Cui^1^, Yu Nie^1^, Ahmad Umar^3, 4^, Qiang Wang^1^,*

^1^College of Environmental Science and Engineering, Beijing Forestry University, 35 Qinghua East Road, Haidian District, Beijing 100083, P. R. China

^2^Faculty of Environmental Science and Engineering, Kunming University of Science and Technology, Kunming, 650500, Yunnan, P. R. China

^3^Department of Chemistry, College of Science and Arts, Najran University, Najran–11001, Kingdom of Saudi Arabia

^4^Promising Centre for Sensors and Electronic Devices (PCSED), Najran University, Najran–11001, Kingdom of Saudi Arabia

*Corresponding author:

Professor Qiang Wang, College of Environmental Science and Engineering, Beijing Forestry University, 35 Qinghua East Road, Haidian District, Beijing 100083, P. R. China

Tel.: 86–13699130626

E–mail: [qiang.wang.ox@gmail.com](mailto:qiang.wang.ox@gmail.com); [qiangwang@bjfu.edu.cn](mailto:qiangwang@bjfu.edu.cn)


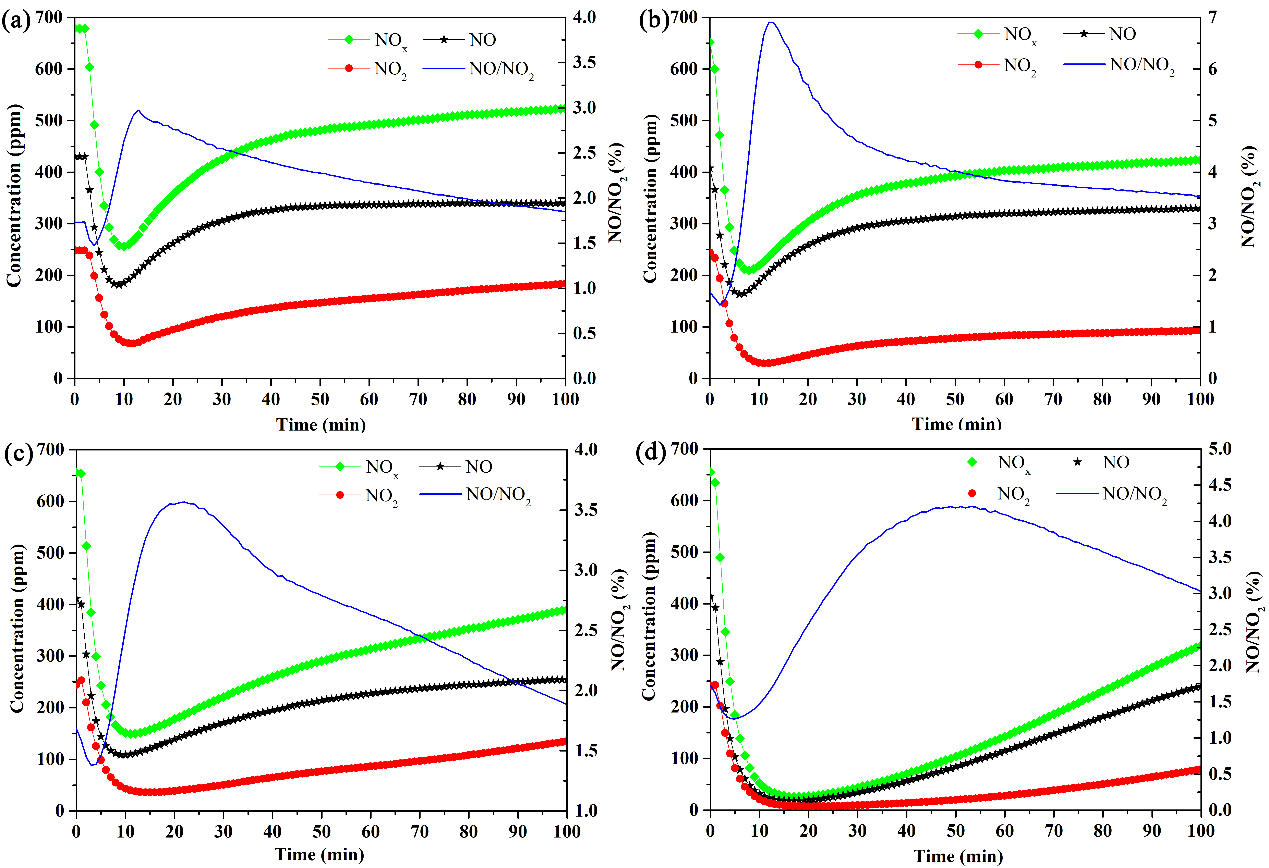


**Fig. S1.** NO_x_, NO_2_, NO concentration, and the ratio of NO/NO_2_ for (a) MgAlO_x_, (b) MgAlO_x_–rGO, (c) 2Pt/10K/MgAlO_x_, and (d) 2Pt/10K/MgAlO_x_–rGO catalysts tested at 350 ^o^C.


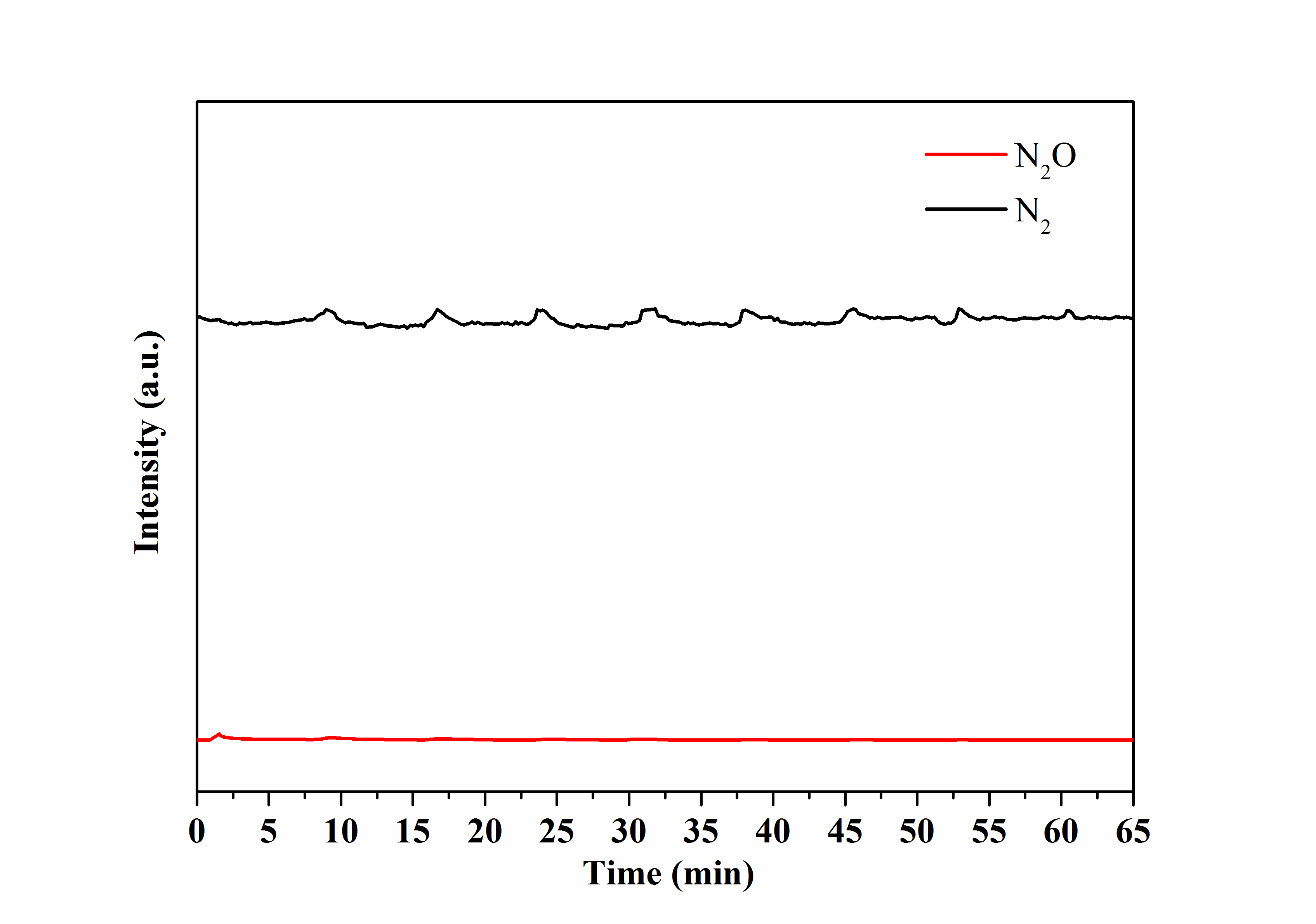


**Fig. S2.** Intensity of effluent N_2_ and N_2_O during lean–rich cycling tests over 2Pt/10K/MgAlO`–rGO catalyst at 350 ^o^C.
